# A hypoglycaemic ‘Peter Pan’: a paediatric disease in an adult patient?

**DOI:** 10.1530/EDM-23-0140

**Published:** 2025-06-09

**Authors:** Shannon McCarthy, Mark Kotowicz

**Affiliations:** Barwon Health, Geelong, Australia

**Keywords:** Paediatric endocrinology, hypoglycaemia, pancreatic disease

## Abstract

**Summary:**

A 56-year-old male presented to hospital with vomiting and was admitted for management of suspected aspiration pneumonia. His medical history was significant for a diagnosis of cerebral palsy and intellectual disability and he had suffered regular generalised tonic–clonic seizures (GTCS) since birth, despite multimodal anticonvulsant treatment. During his admission, his capillary blood glucose was noted to be 1.6 mmol/L during a seizure. Subsequent investigations confirmed hyperinsulinaemic hypoglycaemia secondary to diffuse pancreatic nesidioblastosis. His seizure disorder completely resolved when management of nesidioblastosis achieved consistent normoglycaemia.

**Learning points:**

- All patients who suffer seizure should have a blood glucose measured.
- Unrecognised hypoglycaemia in a neonate or infant confers a high risk of subsequent neurological damage.
- Persistent hyperinsulinaemic hypoglycaemia (PHH) in adults is highly likely to be caused by insulinoma, but diffuse pancreatic hyperinsulinism, particularly after bariatric surgery, should also be considered.
- Medical therapy of endogenous hyperinsulinaemic hypoglycaemia is complex, requiring intensive monitoring.

## Background

Paediatric hyperinsulinaemic hypoglycaemia is well-described in neonates and infants, and unlike adult hyperinsulinaemic hypoglycaemia, is more commonly due to monogenic congenital hyperinsulinism or nesidioblastosis than insulinoma. In adults, endogenous hyperinsulinaemia hypoglycaemia is due to insulinoma in approximately 95% of cases.

This case is unusual as not only does it describe an adult patient with nesidioblastosis outside the post-bariatric surgery setting, but also because it is likely that unrecognised neonatal nesidioblastosis caused lifelong generalised seizures and contributed to significant neurological disability due to hypoglycaemic brain injury.

Management was made even more challenging by our patient’s intellectual disability, his inability to communicate verbally, poor tolerance of medical investigations and interventions, and issues of appropriate medical management, consent, and risk management.

## Case presentation

A 56-year-old Caucasian male patient was admitted under a general medical unit after an episode of persistent nausea and vomiting, complicated by likely aspiration pneumonia.

His management was challenging. As an infant he was macrosomic and was diagnosed with cerebral palsy and was noted to have motor deficits. He suffered from frequent GTCS since infancy, sometimes multiple times daily. His mother reported that he had been reviewed by multiple paediatricians and paediatric neurologists, had blood tests, cerebral imaging and EEG, but she could not recall any specific findings or diagnoses other than cerebral palsy.

He was treated with multiple different anticonvulsants over many years as a child, but seizures still occurred at least once a week and sometimes multiple times daily in adulthood. Seizures were more likely during acute illness, but also occurred spontaneously with no evident trigger (illness, sleep deprivation, bright lights, etc.). Seizures lasted between 10 seconds and 3 minutes and would spontaneously terminate. After trial of multiple different medications and combinations of such over his lifetime, his family were told his condition was refractory and treatment was maintained as sodium valproate 500 mg tid and carbamazepine 600 mg for at least 20 years before this presentation, with the addition of olanzapine greater than 5 years before to manage agitation. Seizures continued and, on medical advice, the family ceased calling the ambulance or seeking acute treatment. There had been no change in seizure frequency for over 20 years before presentation.

He was intellectually disabled and non-verbal throughout his life; his carers, mother and sister provided the required collateral history. He was a tall gentleman and heavy set, his mother stating that he had been taller and heavier than his peers since toddlerhood and had a voracious appetite, eating large meals and communicating hunger between meals and overnight; family and carers were mindful of his portion sizes, but reported he would eat anything left within his reach, and this was also observed whilst inpatient. Blood tests and cannulation required sedation to be performed safely due to aggression.

There was no family history of epilepsy, endocrine disorders, or obesity.

He lived at home with his mother until age 55 years, when he suffered a fall, sustaining a fracture of the left inferior pubic ramus. Before this, he could transfer from bed to chair independently, but could not mobilise independently. He was no longer able to transfer independently and moved into supported accommodation with carers. Aside from the abovementioned, he had no other significant past medical history and family medical history was unremarkable; of note, there was no family history of neuroendocrine tumours or other endocrine disorders.

His medications were paracetamol 1,330 mg tid, sodium valproate 500 mg tid, carbamazepine 600 mg, and olanzapine 5 mg daily.

Three days before admission, the patient was noted to choke on his food and suffered intermittent coughing and vomiting the following day. He was reviewed at his residence by a locum general practitioner (GP), who prescribed oral antibiotics, prednisolone, and prochlorperazine for a suspected lower respiratory tract infection. He went on to develop persistent vomiting and was brought to hospital.

On initial assessment in the emergency department at 01:00 h, he was comfortable at rest. Heart rate was 112 beats per minute and regular, oxygen saturation was 100% on room air, blood pressure was 141/94 mmHg, and Glasgow Coma Scale score was 11 due to decreased verbal communication skills. The patient’s sister stated his level of alertness and interaction was normal for him. Cardiac, respiratory and gastrointestinal examinations were normal within the limited examination that the patient would tolerate and he did not appear clinically dehydrated. He was not weighed at the time, but on visual inspection, he was obese. He was noted to have ankle oedema.

Over the day he continued to vomit and received ondansetron, 4 mg IM, and later metoclopramide, 10 mg IM; when vomiting continued, he received ondansetron, 8 mg sublingually and a further 20 mg IM metoclopramide. On trial of oral medication, he again choked and vomited and became febrile with a tympanic membrane temperature of 38°C. He was admitted, given intramuscular antibiotics, reviewed by the speech pathologist, and it was recommended he be made nil by mouth. He was cannulated and pathology sent; chest and abdominal x-rays were unremarkable.

He was reviewed by the treating medical team at 11:30 h on the day of admission and was stable. The speech pathologist upgraded him to a soft diet at that time. At 12:00 h, he experienced a witnessed, 30 s GTCS, with spontaneous termination. At cessation, BP was 160/84 mmHg, heart rate 92 beats per minute and regular, oxygen saturation 86% on room air. Capillary blood glucose (CBG) was 3.6 mmol/L and 12.5 g IV dextrose was administered. At 14:00 h, CBG was 1.6 mmol/L. The patient was alert and was treated with glucose drinks. At 15:00 h, CBG was 2.0 mmol/L and at 17:00 h, 5.7 mmol/L. At 22:00 h, CBG was 1.9 mmol/L and the patient refused an oral glucose drink. He was reviewed by resident staff and 25 g IV dextrose was administered, with the CBG rising to 10.1 mmol/L.

The following day, he had multiple episodes of hypoglycaemia despite a continuous infusion of 10% IV dextrose at 100 mL/hour and was reviewed by the on-call endocrinologist.

On review of his previous medical records, it was noted that when the patient was reviewed regarding his pelvic fracture in 2012, his CBG was 1.9 mmol/L, improving to a peak of 4.4 mmol/L with 50 mL 50% IV dextrose. In the following 2 h, he had two GTCS lasting 60 s each, spontaneously terminating without intervention, without glucose measurement at the time; it was 4.1 mmol/L at discharge.

## Investigation

A central line was inserted under sedation to facilitate frequent venous blood sampling. The results of the 72 h fast are found in Table 1. 3-Hydroxy butyrate was undetectable, anti-insulin antibodies were negative, and a serum sulphonylurea screen was negative.

**Table 1:** Results of the 72 h fast. The test was terminated prematurely as there was evidence of ongoing hyperinsulinaemic hypoglycaemia, which failed to respond to IV glucagon.

Time	CBG (mmol/L)	Intervention	PBG (mmol/L)	Insulin (mU/L)*	C-peptide (nmol/L)*	Proinsulin (pmol/L)*	GH (mU/L)*
01:00	3.5	Lucozade					
02:00	3.7	Glucose gel + ice cream					
03:00	6.6						
04:00	5.1						
05:00	4.8						
06:00	2.7	Glucose gel					
07:00	3.3	Glucose gel					
08:00	5.3	Glucose gel					
10:05	3.2	Fast commenced	2.6	23.5	0.92	65.1	
11:00	4.2						
12:30				38.8	1.30	54.9	
13:00	1.6		1.4	31.7	1.01	31.5	
14:00	5.2						
15:00	3.2		1.4				
16:00	2.5						
17:05	1.9		1.4	28.1	0.78	26.0	
18:00	6.7						
19:00	2.2						
19:38			1.5	12.4	0.52	28.6	
19:55	3.0		1.5	31.1	0.83	39.3	5.6
20:15		Glucagon 1 mg IV					
20:25			2.8				
20:35			3.0				
20:45			2.3				

CBG, capillary blood glucose; PBG, plasma blood glucose; GH, growth hormone.

* Reference range – insulin: <15.0 mU/L; C-peptide: 0.30–1.30 nmol/L; proinsulin: 13.3 pmol/L; GH: 0.1–12.0 mU/L.

A contrast CT scan of the abdomen and pelvis was performed under general anaesthetic. The pancreas and intestines were normal. There was steatosis of the liver, a 13 mm cyst in the left kidney, a hiatus hernia, bibasal atelectasis, a small amount of pericardial fluid, and a haemangioma in the second lumbar vertebral body.

An abdominal ultrasound was attempted, but the patient failed to tolerate the examination.

An endoscopic ultrasound of the pancreas was performed, demonstrating fatty infiltration of the pancreas, liver, and a small, benign-appearing liver cyst.

## Treatment

Extensive discussion took place with the patient’s mother and sister regarding the appropriate approach to his investigation and management and the decision was made not to persist with further investigation, but to attempt to manage his hypoglycaemia with medical therapy.

He commenced treatment with dexamethasone 1 mg orally daily and diazoxide 100 mg orally three times daily, but continued to have frequent hypoglycaemia even when the dose of diazoxide was rapidly escalated to a total daily dose of 400 mg tid and dexamethasone to 1 mg bd.

Octreotide was added, but he developed significant hyperglycaemia and ketosis, which persisted despite the withdrawal of concurrent diazoxide and dexamethasone. Furthermore, there was a high degree of distress and aggression associated with injections that became unsafe for staff and octreotide was ceased.

The patient began refusing food, fluid, and medication, which was very distressing to his mother, but he improved with empirical antibiotic treatment for presumed aspiration pneumonia. His CBGs stabilised to 3.8–10.8 mmol/L with hydrochlorothiazide 50 mg orally daily.

Treatment was made difficult by the development of hyperglycaemia and ketosis with octreotide; distress when receiving parenteral therapies, undergoing blood tests or attempts at cannulation; intermittent refusal of oral medications and food; and ongoing vomiting and likely aspiration.

## Outcome and follow-up

After the initial admission of 51 days, the patient was discharged to supported care on his pre-admission medications and hydrochlorothiazide 50 mg orally daily. His carers were educated to perform regular CBG monitoring and subsequently contacted the endocrinology unit frequently for advice regarding his ongoing asymptomatic hypoglycaemia.

The patient was readmitted 3 weeks later due to frequent ongoing hypoglycaemia and vomiting episodes, with significant hyponatraemia. After stabilization, he was transferred to a tertiary referral centre for a pancreatic arterial calcium stimulation test. The stimulation revealed a diffuse rise in insulin production throughout all areas of the pancreas, in keeping with nesidioblastosis rather than an insulinoma. Treatment with dexamethasone 1 mg orally daily and diazoxide 100 mg orally twice daily was initiated.

Once this treatment commenced, despite intermittent hypoglycaemia, the patient suffered no further seizures.

Following the diagnosis of nesidioblastosis, the option of curative therapy with pancreatic resection was discussed with his family, who felt that a major surgery would be extremely distressing to him and the risk of subsequent insulin-dependent diabetes mellitus and the implications of its management were unacceptably high. He continued therapy with diazoxide and dexamethasone, which were cautiously increased as an outpatient.

Several months later, the patient was admitted with diabetic ketoacidosis, with a contribution of starvation ketosis. Insulin therapy was initiated and he was stabilised on a regimen of Humalog Mix 50, 44 units subcutaneously with breakfast and 10 units subcutaneously with dinner. Diazoxide was ceased and dexamethasone was substituted with hydrocortisone 10 mg orally mane. His supported accommodation was not equipped to administer insulin and he was discharged to high level care.

Two months later, he was reviewed in endocrinology outpatients and insulin was ceased due to frequent hypoglycaemia.

The patient was readmitted to hospital several months later in a hyperglycaemic hyperosmolar state, complicated by staphylococcal sepsis and candidaemia. His family chose palliative treatment and he passed away soon after, 18 months after his initial admission.

## Discussion

This case demonstrates the importance of some of the difficulties that may be experienced in the diagnosis of hypoglycaemic disorders.

In adults, the most common cause of hyperinsulinaemic hypoglycaemia is insulinoma, accounting for 95% of cases; nesidioblastosis in adults is usually limited to the post-bariatric surgery population, of which our patient was not making his case unusual.

Our patient was born in 1956, well before the development of point-of-care capillary blood glucose monitoring, which would have easily identified hypoglycaemia at the time of a seizure. There is no way of knowing when his hyperinsulinism developed and it is possible that it was persistent from birth. This case does raise a difficult question in the management of chronic disease – when or how often should we re-investigate a refractory or idiopathic condition for secondary causes?

There are multiple case reports of nesidioblastosis and insulinoma not only being initially misdiagnosed as epilepsy, but also migraine or psychiatric conditions (1, 2, 3).

Persistent hyperinsulinaemic hypoglycaemia in neonates is well-recognised and is due to inactivating mutations of the ATP-sensitive potassium channels in the beta-cell membrane, or less commonly, gain-of-function mutations of glucokinase or glutamate dehydrogenase. Congenital hyperinsulinism occurs in 1/50,000 live births and has a high risk of causing seizures and intellectual disability (4). However, the clinical presentation and severity of disease are heterogeneous; up to 25% of patients go on to develop T2DM (5, 6).

## Declaration of interest

The authors declare that there is no conflict of interest that could be perceived as prejudicing the impartiality of the work reported.

## Funding

This work did not receive any specific grant from any funding agency in the public, commercial or not-for-profit sector.

## Patient consent

Every effort was made to contact the next of kin of the deceased patient to obtain consent but was unsuccessful.

## Author contribution statement

SM wrote the first draft of this manuscript, which was then reviewed by MK, who edited and finalised the version.
